# Semaglutide Attenuates Inflammatory Macrophage Polarization and Promotes Changes Associated with Mitochondrial Biogenesis Through cAMP-Dependent Signaling

**DOI:** 10.3390/biomedicines14091987

**Published:** 2026-09-03

**Authors:** Vsevolod V. Pavshintsev, Aleksandra Zh. Erbaeva, Aleksey A. Vatlin, Nikita A. Mitkin

**Affiliations:** 1 Laboratory of Angiopathology, Institute of General Pathology and Pathophysiology, 8 Baltiiskaya Street, 125315 Moscow, Russia; 2 Institute of Environmental Engineering, RUDN University, 6 Miklukho-Maklaya Street, 117198 Moscow, Russia

**Keywords:** semaglutide, GLP-1 receptor, cAMP signaling, macrophage polarization, mitochondrial biogenesis, metabolic inflammation

## Abstract

**Background/Objectives**: Chronic metabolic inflammation is associated with pro-inflammatory macrophage activation and mitochondrial dysfunction. Semaglutide, a widely used GLP-1 receptor agonist, has been reported to possess systemic anti-inflammatory properties, but its action on human macrophages, especially in the context of mitochondrial regulation, remains poorly understood. The aim of the study was to evaluate the direct effect of semaglutide on macrophage polarization and to investigate the roles of cAMP signaling and mitochondrial regulation in this process. **Methods**: THP-1 and U-937 monocytic cell lines were differentiated into macrophages and polarized into the M1-like phenotype using LPS/IFN-γ. Cells were treated with 100 nM semaglutide in the presence or absence of the adenylyl cyclase inhibitor SQ22536. Intracellular cAMP levels, mRNA expression of macrophage markers and key regulators of mitochondrial biogenesis (SIRT1 and PGC-1α), and levels of pro- and anti-inflammatory cytokines were determined. The COX-1/SDH-A and mtDNA/nDNA ratios were analyzed as markers of mitochondrial biogenesis. **Results**: Semaglutide induced cAMP accumulation in M0- and M1-like macrophages derived from both cell lines. This response was significantly attenuated by GLP-1R antagonist exendin(9–39), supporting the presence of functional GLP-1R signaling. Semaglutide supplementation during inflammatory polarization resulted in reduced levels of pro-inflammatory markers (CD80/CD86, TNF-α, IL-6) and increased expression of M2-associated genes CD206 and CD163 and secretion of the anti-inflammatory cytokine IL-10. Semaglutide also increased SIRT1 and PGC-1α expression, the COX-1/SDH-A ratio, and the mtDNA/nDNA ratio, suggesting activation of processes associated with mitochondrial biogenesis. All of the effects of semaglutide described above were attenuated by SQ22536, supporting an important contribution of cAMP signaling to these responses. **Conclusions**: Semaglutide acts directly on human monocytic cell line-derived macrophages, attenuating the M1-like program and promoting a shift toward a less inflammatory phenotype. These effects strongly depend on cAMP signaling and are accompanied by increased expression of SIRT1 and PGC-1α and mitochondrial changes consistent with enhanced biogenesis.

## 1. Introduction

Chronic low-grade inflammation is a hallmark of metabolic syndrome and related conditions such as obesity, insulin resistance, and cardiometabolic diseases. Macrophages have been implicated in driving the process due to their accumulation in the expanded adipose tissue and transition toward a pro-inflammatory phenotype that contributes to increased production of inflammatory cytokines and impaired insulin sensitivity [1]. The balance between pro-inflammatory and reparative macrophage populations is influenced by cellular metabolism: pro-inflammatory macrophages primarily rely on glycolysis, whereas reparative ones depend on mitochondrial oxidative phosphorylation for energy [2]. Therefore, macrophage activation is closely linked to insulin sensitivity, lipid metabolism, and mitochondrial homeostasis.

Glucagon-like peptide-1 receptor agonists (GLP-1RAs) are associated with effects beyond glucose regulation. In the SELECT trial, semaglutide improved cardiovascular outcomes, including major adverse cardiovascular events, in overweight/obese patients without diabetes [3]. In a series of preclinical studies it was shown that GLP-1 or GLP-1RAs can attenuate macrophage infiltration into adipose tissue, suppress inflammatory cytokine expression, and reduce atherosclerosis development [4,5,6]. However, while anti-inflammatory properties of semaglutide are well established at a systemic level, the exact cellular mechanisms remain poorly understood. The analysis of samples from STEP 1 and STEP 2 trials showed that semaglutide downregulated multiple circulating inflammatory and immune response-related proteins independently of weight loss and glycemic improvement [7]. At the same time, the peripheral immune cell populations responsible for the response have not been identified. Specifically, in mice, the acute inhibition of Toll-like receptor agonist-induced TNF-α in response to GLP-1RA was mediated by neuronal, rather than hematopoietic, GLP-1Rs [8]. It should also be noted that the effects of GLP-1RAs on inflammation are context-dependent. While in atherosclerosis models liraglutide skews macrophage polarization towards an anti-inflammatory M2-like profile [5], in papillary thyroid carcinoma semaglutide was shown to drive tumor-associated macrophages toward an M1 phenotype by remodeling lipid metabolism through a proposed GLP-1R/PPARG/ACSL1 axis [9].

GLP-1R is a GPCR that couples to the stimulatory G protein alpha subunit (Gαs) [10]. Upon ligand binding, the Gαs subunit activates adenylyl cyclase, leading to increased intracellular levels of cAMP. The cAMP signaling pathway is known to play a key role in the function of GLP-1, semaglutide, and other GLP-1RAs [11]. Semaglutide-induced cAMP signaling has been mostly studied in hindbrain neurons, where it mediates the effects of the drug on food intake and body weight [12]. Regarding mitochondrial function, semaglutide improves mitochondrial oxidative phosphorylation efficiency in skeletal muscle during weight loss [13] and regulates glutamine metabolism by inducing muscle mitochondria [14]. Human circulating polymorphonuclear leucocytes also respond to treatment with a GLP-1RA by enhancing mitochondrial respiration [15]. However, neither observation establishes a causal relationship between mitochondrial function and cAMP production in macrophages. Moreover, the expression of functional GLP-1R in myeloid cells has been debated, with a recent study showing that it is variable across different models [8]. Therefore, it remains unclear whether semaglutide can directly impact human macrophage immunometabolism through cAMP and whether this engages mitochondrial regulatory pathways.

Although the polarization of monocytic cell lines into M1/M2 macrophages is an experimental simplification of the complex spectrum of immunologic responses, it serves as a useful model to study the regulation of inflammatory processes. In the present study, we investigate whether semaglutide can modulate the LPS/IFN-γ-induced inflammatory polarization of macrophages obtained from monocytic cell lines via cAMP-dependent mechanisms and regulate mitochondrial biogenesis.

## 2. Materials and Methods

### 2.1. Cell Lines

THP-1 human monocytic leukemia cell line and U-937 human pro-monocytic lymphoma cell line were obtained from the Collection of Cell Cultures of Vertebrates, Institute of Cytology RAS (Saint Petersburg, Russia). Both cell lines were validated by short tandem repeat (STR) profiling performed prior to the experiments. THP-1 and U-937 cells were cultivated as suspension cultures in RPMI-1640 medium (Paneco, Moscow, Russia) supplemented with 10% fetal bovine serum (Thermo Scientific, Waltham, MA, USA) at 37 °C, 5% CO_2_ in a humidified tissue culture incubator. THP-1- and U-937-derived macrophages were maintained as adherent cell cultures under the same conditions.

### 2.2. Macrophage Differentiation and Polarization into an M1-like Phenotype

Differentiation into macrophages of the neutral M0 phenotype was induced by incubation for 24 h with 80 ng/mL and 50 ng/mL of PMA (Sigma-Aldrich, Burlington, MA, USA) for THP-1 [16] and U-937 [17], respectively. Differentiation efficiency was monitored by the progressive transition of the cells from a suspension to an adherent state.

Polarization into an inflammatory M1-like phenotype was performed by incubation of M0-like cells with 10 ng/mL LPS (Sigma-Aldrich, Burlington, MA, USA) and 20 ng/mL IFN-γ (GenScript, Nanjing, China) for 24 h. Preliminary experiments with various concentrations of LPS (0.01, 0.1, 1, 10, and 100 ng/mL) revealed 10 ng/mL as the most effective dose for both THP-1 and U-937, resulting in the strongest increase in CD80 gene expression (M1-specific marker), as measured by real-time PCR, while maintaining high cell viability. Therefore, this treatment regimen, which also corresponds to published protocols [18,19], was chosen for all subsequent experiments.

### 2.3. Selection of Semaglutide and SQ22536 Concentrations and HTRF cAMP Assay

Both M0-like macrophages after PMA priming and M1-like macrophages after LPS/IFN-γ-induced polarization, derived from THP-1 and U-937, were treated with different semaglutide (Cayman Chemical, Ann Arbor, MI, USA) concentrations (0.001, 0.01, 0.1, 1, 10, 10^2^, 10^3^, 10^4^ nM) followed by measurement of intracellular cAMP levels using HTRF cAMP Gs HiRange Detection Kit (Revvity, Waltham, MA, USA) according to the manufacturer’s protocol. Briefly, 5 µL of cell suspension was seeded at a density of 3000 cells/well in a 384-well plate. Cells were treated with 5 µL of semaglutide solution at 37 °C for 30 min. 5 µL cAMP-d2 working solution and anti-cAMP-Cryptate working solution were added to the cells and incubated for 60 min at RT. The signal of the assay plate was detected using an HTRF-compatible reader PerkinElmer EnVision (PerkinElmer, Waltham, MA, USA). The ratio of the acceptor and donor emission signal for each well was calculated using the following formula: Ratio = Signals_665nm_/Signals_620nm_ × 10^4^. Mean HTRF ratio at the top concentration of semaglutide (10^4^ nM) was taken as 100% stimulation. %Stimulation for other concentrations was calculated according to the formula: %Stimulation = (Mean HTRF ratio at tested concentration − Mean HTRF ratio without semaglutide)/(Mean HTRF ratio at top concentration − Mean HTRF ratio without semaglutide) × 100%. Percentage stimulation was plotted against semaglutide concentration. Semaglutide concentration-response curves were fitted by nonlinear regression using a four-parameter logistic model in GraphPad Prism version 8.0.1, and EC50 values and Hill slopes were calculated for each macrophage model.

To assess the involvement of GLP-1R in semaglutide-induced cAMP accumulation, M0- and M1-like macrophages derived from THP-1 and U-937 cells were treated with the GLP-1R antagonist exendin(9–39) (Thermo Scientific, Waltham, MA, USA) at final concentrations of 1 and 3 μM, 20 min prior to stimulation with the selected concentration of semaglutide. Exendin(9–39) was maintained in the medium during semaglutide stimulation, and intracellular cAMP levels were subsequently determined using the HTRF assay described above. Mean HTRF ratio in the absence of exendin(9–39) was taken as 100% stimulation. %Stimulation for other samples was calculated according to the formula: %Stimulation = (Mean HTRF ratio at tested exendin(9–39) concentration − Mean HTRF ratio without semaglutide)/(Mean HTRF ratio without exendin(9–39) − Mean HTRF ratio without semaglutide) × 100%.

To select an effective concentration of the adenylyl cyclase inhibitor SQ22536, the cells seeded in a 384-well plate were treated with a combination of semaglutide at the selected concentration (100 nM) and different concentrations of SQ22536 (Sigma-Aldrich, Burlington, MA, USA): 2, 20, 200, 2000 μM. All other procedures were performed as described above. Mean HTRF ratio in the absence of SQ22536 was taken as 100% stimulation. %Stimulation for other samples was calculated according to the formula: %Stimulation = (Mean HTRF ratio at tested SQ22536 concentration − Mean HTRF ratio without semaglutide)/(Mean HTRF ratio without SQ22536 − Mean HTRF ratio without semaglutide) × 100%. Percentage stimulation was plotted versus SQ22536 concentrations. The data were fitted by nonlinear regression using a variable-slope inhibitor-response model in GraphPad Prism. The 0 μM SQ22536 condition was included in the regression analysis as the 100% stimulation reference, and IC50 values were calculated for each macrophage model.

### 2.4. RNA Extraction, cDNA Synthesis and Real-Time Quantitative RT-PCR

Cells were collected for RNA extraction 24 h after the initiation of M1 polarization (following the addition of LPS/IFN-γ with or without 100 nM semaglutide and 200 μM SQ22536). Total RNA was isolated using ExtractRNA reagent (Evrogen, Moscow, Russia) according to the manufacturer’s protocol, followed by cDNA synthesis using 2 μg of total RNA from each sample and the MMLV RT kit (Evrogen, Moscow, Russia) with oligo-dT primers.

Quantitative RT-PCR was performed using a CFX96 Touch real-time PCR detection system (Bio-Rad Laboratories, Hercules, CA, USA), qPCRmix-HS SYBR (Evrogen, Moscow, Russia), and specific primers designed to amplify intron-spanning fragments of human ACTB, CD80, CD86, CD206, CD163, SIRT1, PGC-1α, and GLP-1R genes (Supplementary Table S1). ROX was included as a passive reference dye. No-template controls, in which cDNA was replaced with ddH_2_O, were included in each run to control for reagent contamination and nonspecific amplification. Each biological sample was analyzed in three technical replicates. The PCR program included an initial preheating stage at 95 °C for 10 min, followed by 40 cycles of amplification at 95 °C for 15 s, 63 °C for 20 s, and 72 °C for 20 s. The specificity of amplification products was assessed by melting curve analysis, which demonstrated a single amplification product for each primer pair.

Relative gene expression was determined using the comparative Ct method. ΔCt was calculated as Ct (target gene) − Ct (ACTB). The mean ΔCt of the corresponding control group was used as the reference value for calculation of ΔΔCt, and ΔΔCt was calculated as ΔCt (sample) − mean ΔCt (control). Relative expression was expressed as 2^(−ΔΔCt)^, with the mean value of the control group set to 1.

### 2.5. Measurement of Cytokine Levels by ELISA

Conditioned medium was collected 24 h after the initiation of M1 polarization (an addition of LPS/IFN-γ with or without 100 nM semaglutide and 200 μM SQ22536), centrifuged at 500 *g* for 10 min at 4 °C, aliquoted, and stored at –80 °C. The levels of pro-inflammatory cytokines TNF-α, IL-1β, IL-6, and anti-inflammatory cytokine IL-10 were measured using the respective DuoSet ELISA Development kits (R&D Systems, Minneapolis, MN, USA) according to the manufacturer’s protocols.

### 2.6. Assessment of Mitochondrial Biogenesis-Associated Changes

Twenty-four hours after the initiation of polarization, the culture medium of THP-1-derived M1-like macrophages treated with or without semaglutide and SQ22536 was replaced with fresh medium containing the corresponding compounds at final concentrations of 100 nM semaglutide and 200 μM SQ22536. The cells were then cultured for an additional 4 days, with daily medium replacement. At the end of the cultivation period, mitochondrial biogenesis-associated changes were evaluated using two complementary approaches: determination of the COX-1/SDH-A protein ratio and assessment of relative mitochondrial DNA (mtDNA) content.

The COX-1/SDH-A ratio was determined using the Colorimetric MitoBiogenesis In-Cell ELISA Kit (Abcam, Cambridge, UK) according to the manufacturer’s instructions. The assay quantifies the mitochondrial DNA-encoded cytochrome C oxidase subunit 1 (COX-1, a subunit of respiratory complex IV) and the nuclear DNA-encoded succinate dehydrogenase subunit A (SDH-A, a subunit of respiratory complex II) proteins, with the COX-1/SDH-A ratio used as an indirect indicator of mitochondrial biogenesis.

For assessment of relative mtDNA content, total cellular DNA was isolated from parallel cell samples using the Quick-DNA Plus Kit (Zymo Research, Irvine, CA, USA) according to the manufacturer’s protocol. Relative mtDNA content was determined by quantitative real-time PCR by comparing the level of the mtDNA-encoded MT-ND1 locus with that of the nuclear DNA-encoded reference gene B2M as described earlier [20]. Quantitative real-time PCR was performed applying CFX96 Touch real-time PCR detection system (Bio-Rad Laboratories, Hercules, CA, USA), qPCRmix-HS SYBR (Evrogen, Moscow, Russia), and specific primers listed in Supplementary Table S1. For each sample, ΔCt was calculated as Ct (MT-ND1) − Ct (B2M). Relative mtDNA content was calculated as 2^(−ΔCt)^.

### 2.7. MTT-Based Assessment of Metabolic Cell Viability

Viability was assessed under both the short-term treatment conditions used for macrophage polarization experiments and the prolonged cultivation conditions used for analysis of mitochondrial biogenesis-associated parameters. At the corresponding experimental endpoint, the culture medium was removed, the cells were washed with PBS and incubated with thiazolyl blue tetrazolium bromide (MTT, Sigma-Aldrich, Burlington, MA, USA) at a final concentration of 0.5 mg/mL in serum-free RPMI-1640 medium (100 μL per well) for 4 h at 37 °C. The MTT-containing medium was removed, and the resulting formazan crystals were dissolved by incubation with 100 μL of acidified isopropanol (0.08 N HCl) for 15 min. Absorbance was measured at 570 nm using a microplate reader. Cell viability was calculated as the percentage of the absorbance measured in M1-like cells relative to the M0-like cells. The mean viability of the M0-like cells was therefore defined as 100%.

### 2.8. Statistical Analysis

Statistical analyses were performed using Microsoft Excel and GraphPad Prism. Differences between groups were evaluated using a one-way ANOVA with post-hoc Tukey multiple-comparison test, with the analysis performed separately for each cell line. A *p*-value < 0.05 was considered statistically significant. Data are presented as the mean ± standard error of the mean (SEM).

## 3. Results

### 3.1. GLP-1R Expression and Pharmacological Antagonism Support the Presence of Functional GLP-1R in M0- and M1-like Macrophages Derived from Monocytic THP-1 and U-937 Cell Lines

We performed a dose–response study to characterize semaglutide-induced cAMP signaling in THP-1- and U-937-derived macrophages and to select a concentration for subsequent experiments. Both PMA-differentiated M0-like macrophages and LPS/IFN-γ-polarized M1-like macrophages were treated with a broad range of semaglutide concentrations, followed by measurement of intracellular cAMP accumulation.

**Figure 1:**
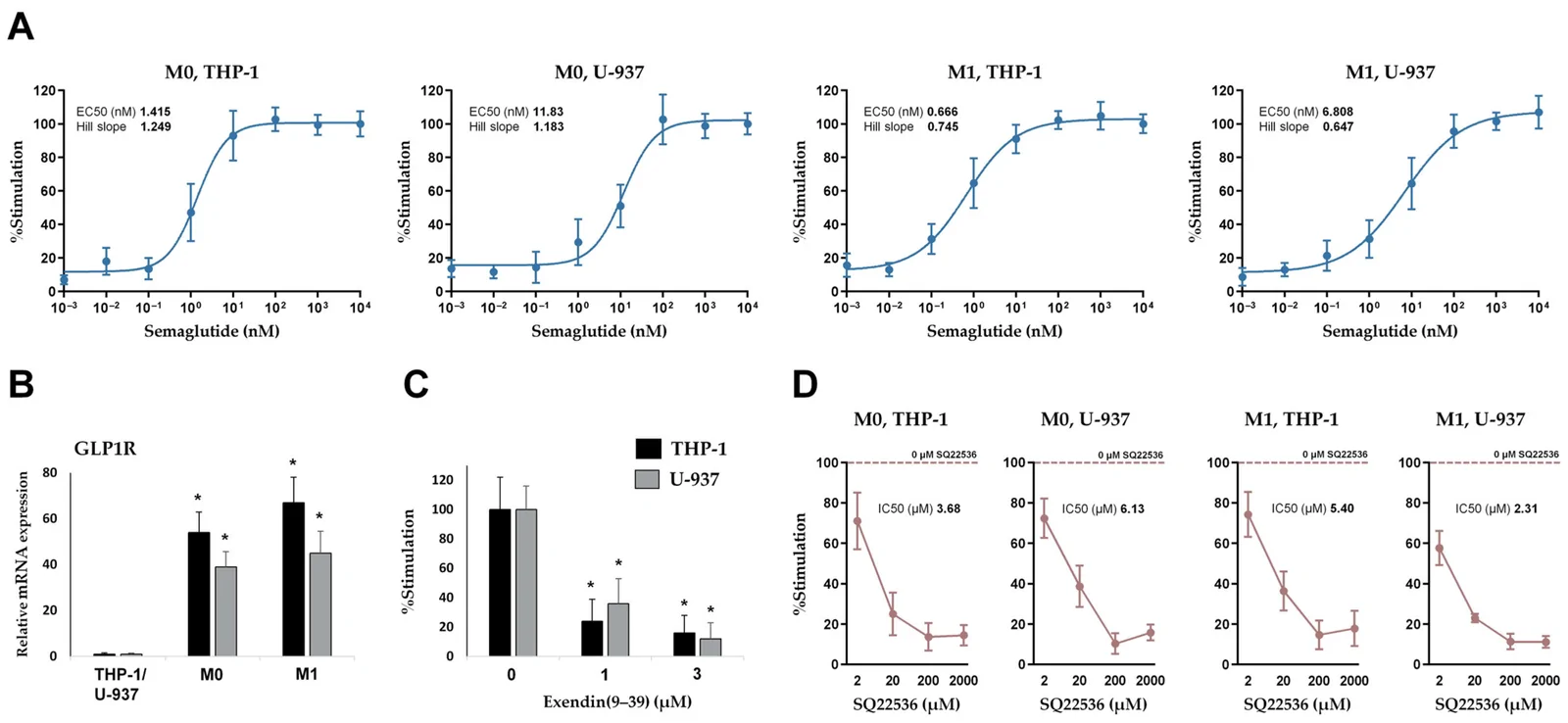
GLP-1R expression and pharmacological characterization of semaglutide-induced cAMP accumulation in M0- and M1-like macrophages derived from THP-1 and U-937 monocytic cell lines. THP-1 and U-937 monocytic cells were differentiated into adherent M0-like macrophages by incubation with PMA. To obtain M1-like macrophages, PMA-differentiated cells were subsequently stimulated with 10 ng/mL LPS and 20 ng/mL IFN-γ for 24 h. (**A**) M0- and M1-like macrophages derived from THP-1 and U-937 cells were treated with semaglutide at concentrations ranging from 0.001 to 10,000 nM for 30 min at 37 °C. Intracellular cAMP levels were measured using an HTRF-based assay. The results are represented as percentage stimulation (%Stimulation), with the response obtained at 10,000 nM semaglutide defined as 100%. Each point represents the mean ± SEM of three independent experiments. Concentration-response curves were fitted using a four-parameter nonlinear regression model with variable Hill slope. (**B**) Relative GLP1R mRNA expression in parental THP-1 and U-937 cells and in M0- and M1-like macrophages derived from the corresponding cell lines. Relative mRNA expression levels were determined by real-time RT-PCR. The data were normalized to β-actin and further normalized to the means for parental THP-1 and U-937 cells (the means for THP-1 and U-937 are represented as 1). Data are presented as the mean ± SEM (three independent experiments). * *p* < 0.05 compared with the THP-1 and U-937 groups, as calculated using a one-way ANOVA with post-hoc Tukey test. (**C**) Effect of the GLP-1R antagonist exendin(9–39) on semaglutide-induced intracellular cAMP accumulation. M0- and M1-like macrophages derived from THP-1 and U-937 cells were treated with 1 or 3 μM exendin(9–39) 20 min before stimulation with 100 nM semaglutide, followed by measurement of intracellular cAMP using the HTRF-based assay. The results are represented as percentage stimulation (%Stimulation), with the response obtained at 100 nM semaglutide without exendin(9–39) defined as 100%. Each point represents the mean ± SEM of three independent experiments. * *p* < 0.05 compared with the control group without exendin(9–39), as calculated using a one-way ANOVA with post-hoc Tukey test. (**D**) Concentration-dependent inhibition of semaglutide-induced cAMP accumulation by the adenylyl cyclase inhibitor SQ22536. M0- and M1-like macrophages derived from THP-1 and U-937 cells were treated simultaneously with 100 nM semaglutide and SQ22536 at concentrations of 2, 20, 200, or 2000 μM. After 30 min of incubation at 37 °C, intracellular cAMP levels were measured using an HTRF-based assay. The results are represented as percent of stimulation (%Stimulation), with the response induced by 100 nM semaglutide in the absence of SQ22536 defined as 100% stimulation. The response to 100 nM semaglutide in the absence of SQ22536 (0 µM SQ22536) is indicated by the horizontal dotted line. The 0 µM SQ22536 condition was included in the nonlinear regression analysis, and IC50 values were calculated using a variable-slope inhibitor-response model. Each point represents the mean ± SEM of three independent experiments.

As shown in Figure 1A, semaglutide induces concentration-dependent increases in cAMP levels in both M0- and M1-like macrophages derived from either THP-1 or U-937 cells, producing characteristic sigmoidal dose–response curves. The lower estimated EC50 values observed for THP-1-derived macrophages indicate greater sensitivity to semaglutide compared with U-937-derived macrophages. Within each cell line, M0- and M1-like macrophages demonstrated broadly comparable concentration-response characteristics, suggesting that inflammatory polarization does not markedly alter semaglutide responsiveness under the experimental conditions used. Among the concentrations tested, 100 nM is the lowest concentration consistently falling within the upper plateau of the cAMP dose–response curves in M0- and M1-like macrophages derived from both THP-1 and U-937 cells. We therefore selected it for subsequent experiments to achieve a robust near-maximal cAMP response across all four macrophage models while avoiding the use of higher concentrations.

GLP1R gene expression levels were evaluated in parental THP-1 and U-937 cells and in M0- and M1-like macrophages derived from both cell lines (Figure 1B). GLP1R expression is relatively low in the parental monocytic cells, with mean Ct values of 34.06 ± 1.08 for THP-1 and 35.08 ± 0.78 for U-937, consistent with THP-1 and U-937 transcriptome profiles from the Cancer Cell Line Encyclopedia (CCLE) [21]. Differentiation into M0-like macrophages significantly increases GLP-1R expression, with mean Ct values decreasing to 27.52 ± 0.23 in THP-1-derived and 29.78 ± 0.22 in U-937-derived macrophages. GLP-1R expression remains elevated after subsequent M1-like polarization, with corresponding mean Ct values of 27.44 ± 0.39 and 29.26 ± 0.17, respectively. These findings demonstrate reproducible detection of GLP-1R transcripts in THP-1- and U-937-derived macrophages. To obtain receptor-specific pharmacological evidence for functional GLP-1R signaling, we evaluated the effect of the GLP-1R antagonist exendin(9–39) on semaglutide-induced cAMP accumulation. Treatment with 1 and 3 μM exendin(9–39) significantly attenuates the cAMP response induced by 100 nM semaglutide in M0- and M1-like macrophages (Figure 1C). Together with the GLP1R expression data, these results indicate the presence of functional GLP-1R-mediated cAMP signaling in the macrophage models used in the study.

SQ22536, a potent cell-permeable, non-competitive inhibitor of adenylyl cyclase [22] reported to block downstream GLP-1R action by reducing cAMP accumulation [23], was tested in the same cell models in an inhibitor mode (Figure 1D). Accumulation of cAMP induced by 100 nM semaglutide is efficiently inhibited by SQ22536, with the maximum effect starting from 20–200 μM for all variants of macrophages tested. A concentration of 200 μM was therefore selected for subsequent experiments.

### 3.2. Semaglutide Attenuates M1-like Macrophage Polarization and Promotes a Shift Toward a Less Inflammatory Phenotype Through cAMP-Dependent Signaling

**Figure 2:**
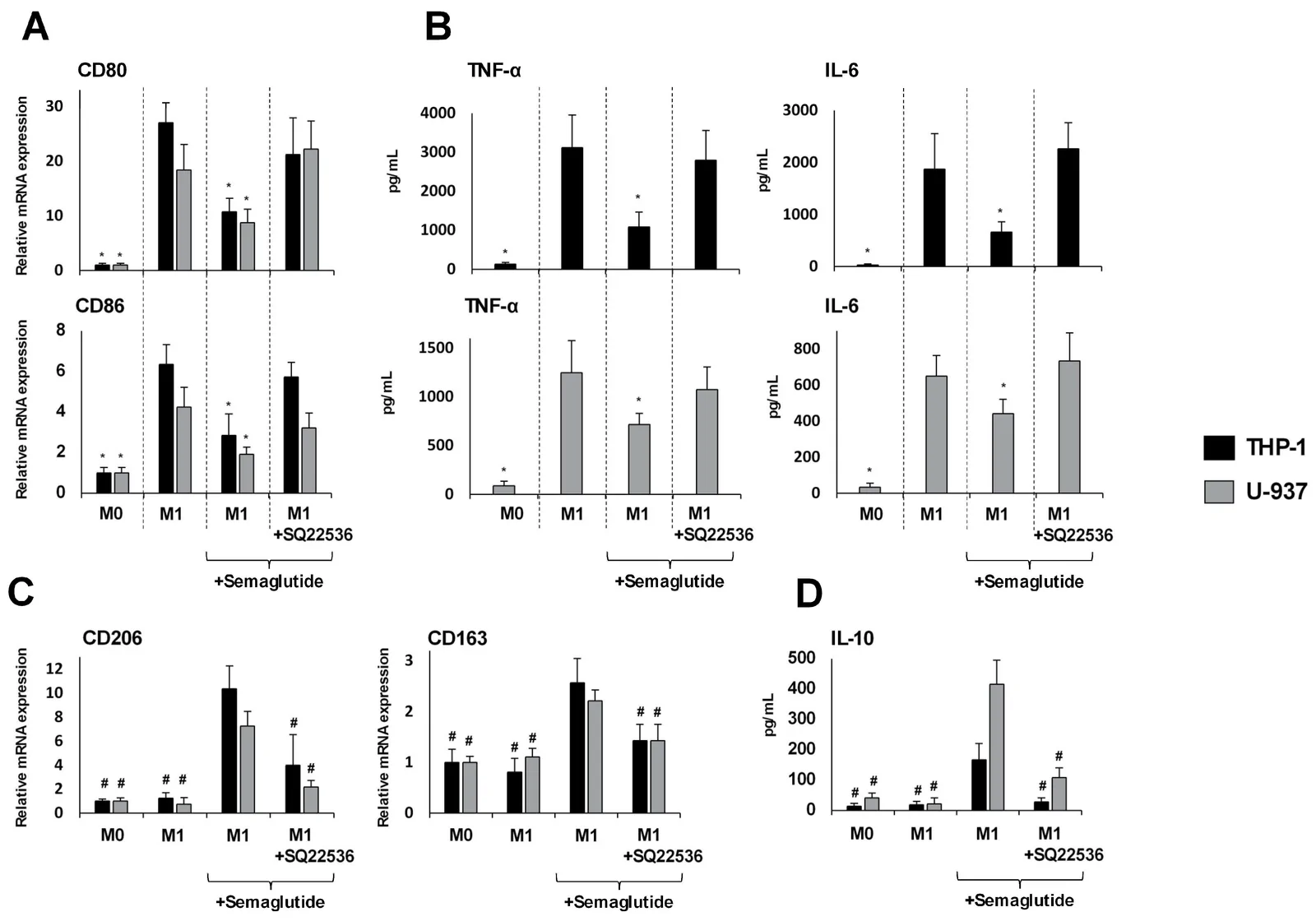
Semaglutide attenuates LPS/IFN-γ-induced M1-like polarization of THP-1- and U-937-derived macrophages and promotes a shift toward a less inflammatory phenotype in a cAMP-dependent manner. THP-1 and U-937 cells were differentiated into M0-like macrophages by PMA treatment and then polarized into the M1-like phenotype by incubation with 10 ng/mL LPS and 20 ng/mL IFN-γ for 24 h in the presence or absence of 100 nM semaglutide and 200 μM SQ22536. (**A**) M1-like polarization results in increased expression of M1 marker genes CD80 and CD86. Semaglutide attenuates the expression of both genes. The addition of SQ22536 attenuates this effect, indicating the involvement of cAMP signaling. Relative mRNA expression levels were determined by real-time RT-PCR. The data were normalized to β-actin and further normalized to the mean for M0 (the means for M0 are represented as 1). Data are presented as the mean ± SEM (five independent experiments). * *p* < 0.05 compared with the untreated M1 group, as calculated using a one-way ANOVA with post-hoc Tukey test. (**B**) M1-like polarization leads to increased secretion of the pro-inflammatory cytokines TNF-α and IL-6. Semaglutide reduces their levels, an effect that is attenuated by SQ22536. Cytokine concentrations in conditioned medium were measured by ELISA. Data are presented as the mean ± SEM (five independent experiments). * *p* < 0.05 compared with the untreated M1 group, as calculated using a one-way ANOVA with post-hoc Tukey test. (**C**) Semaglutide promotes mRNA expression of the M2-associated marker genes CD206 and CD163, indicating induction of an M2-associated transcriptional profile. This stimulatory effect is prevented by SQ22536. Relative mRNA expression levels were measured by real-time RT-PCR. The data were normalized to β-actin and further normalized to the mean for M0 (the means for M0 are represented as 1). Data are presented as the mean ± SEM (five independent experiments). # *p* < 0.05 compared with the M1 + semaglutide group, as calculated using a one-way ANOVA with post-hoc Tukey test. (**D**) Semaglutide increases secretion of anti-inflammatory cytokine IL-10 in both macrophage models, while inhibition of adenylyl cyclase attenuates this effect. IL-10 levels in conditioned medium were determined by ELISA. Data are presented as the mean ± SEM (five independent experiments). # *p* < 0.05 compared with the M1 group treated with semaglutide, as calculated using a one-way ANOVA with post-hoc Tukey test.

In the absence of semaglutide, stimulation of M0 macrophages with LPS/IFN-γ induces M1 polarization, as evidenced by increased expression of the M1-associated markers CD80 and CD86 (Figure 2A) and elevated production of pro-inflammatory cytokines TNF-α and IL-6 (Figure 2B). We did not observe a statistically significant increase in IL-1β secretion in either THP-1- or U-937-derived macrophages (Supplementary Figure S1). This may reflect the strong dependence of IL-1β maturation and release on NLRP3 inflammasome activation and the requirement for excess extracellular ATP in the culture medium as an additional activation signal [24,25]. Semaglutide treatment significantly attenuates the induction of these markers, indicating effective suppression of the pro-inflammatory polarization program. In U-937-derived macrophages, the effect is less pronounced than in THP-1-derived macrophages but, with the exception of IL-6, remains statistically significant, most likely reflecting their lower sensitivity to semaglutide, as demonstrated in the dose–response experiment.

Notably, semaglutide also increases the expression of the M2-associated markers CD206 and CD163 (Figure 2C), as well as the production of the anti-inflammatory cytokine IL-10 (Figure 2D). These findings suggest that semaglutide not only suppresses an M1-like inflammatory program but also promotes a shift toward a less inflammatory phenotype.

The effects of semaglutide on the evaluated markers were significantly attenuated by SQ22536, supporting an important contribution of cAMP signaling to these responses.

To exclude the possibility that SQ22536 influences macrophage polarization independently of semaglutide, we performed an additional control experiment including an SQ22536-only treatment group. SQ22536 alone does not significantly affect expression of macrophage-associated genes or secretion of pro- and anti-inflammatory cytokines compared with untreated M1-like macrophages in either cell line model (Supplementary Figure S2A,B), indicating that it has no detectable effect under these conditions in the absence of semaglutide-induced signaling.

### 3.3. Semaglutide Attenuates the Reduction in SIRT1 and PGC-1α Expression During Pro-Inflammatory Macrophage Polarization

**Figure 3:**
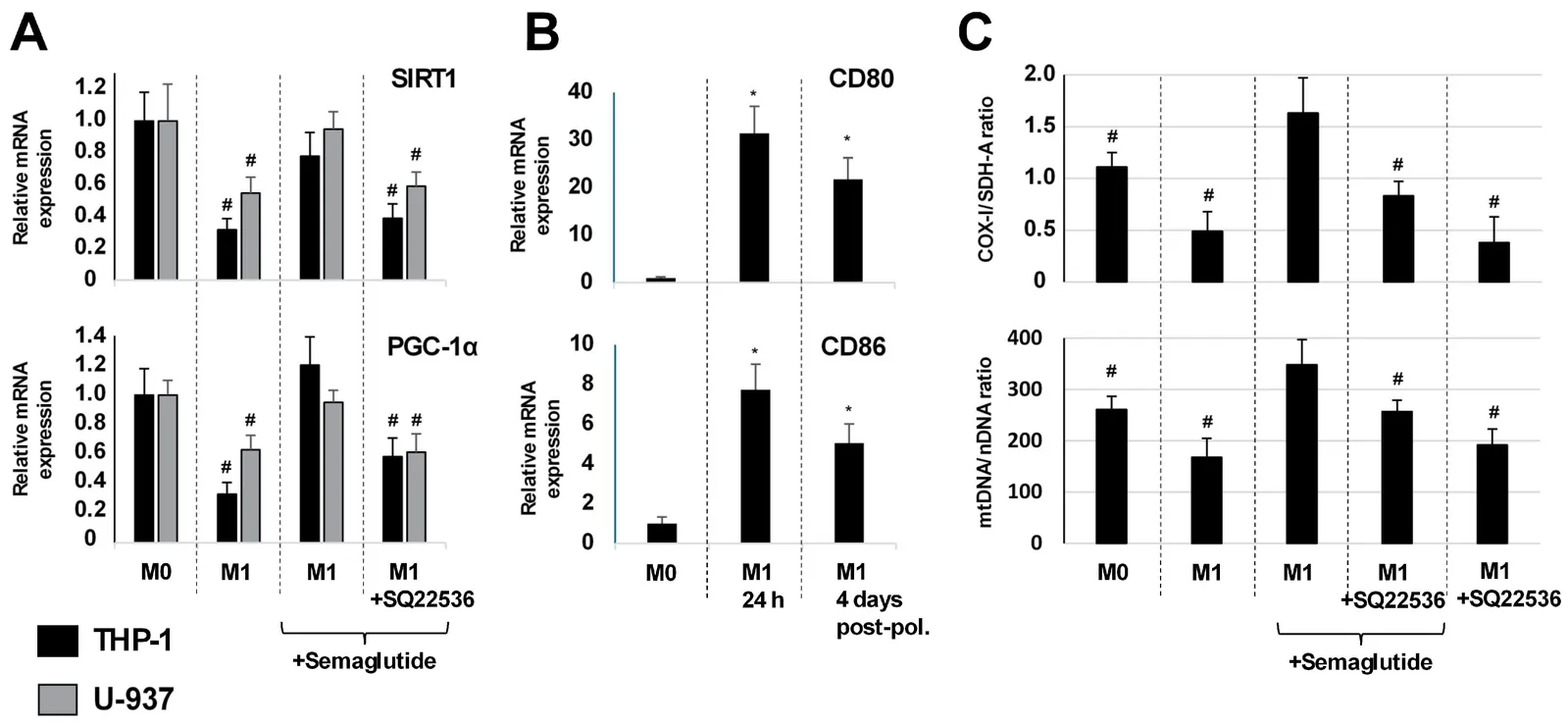
Semaglutide induces the expression of key mitochondrial biogenesis regulators SIRT1 and PGC-1α and promotes mitochondrial changes consistent with enhanced biogenesis in M1-like macrophages through a cAMP-dependent mechanism. (**A**) Relative mRNA expression levels of SIRT1 and PGC-1α in THP-1- and U-937-derived M0-like macrophages and in macrophages polarized with LPS/IFN-γ for 24 h in the presence or absence of 100 nM semaglutide and 200 μM SQ22536. Gene expression levels were determined by real-time RT-PCR. The data were normalized to β-actin and further normalized to the mean for M0 (the means for M0 are represented as 1). Data are presented as the mean ± SEM (five independent experiments). # *p* < 0.05 compared with the M1 group treated with semaglutide, as calculated using a one-way ANOVA with post-hoc Tukey test. (**B**) Relative mRNA expression levels of CD80 and CD86 genes in THP-1-derived M0-like macrophages, M1-like macrophages collected immediately after 24-h LPS/IFN-γ polarization, and M1-like macrophages cultured in the absence of LPS/IFN-γ for an additional 4 days after polarization (M1 4 days post-pol). Gene expression levels were determined by real-time RT-PCR. The data were normalized to β-actin and further normalized to the mean for M0 (the means for M0 are represented as 1). Data are presented as the mean ± SEM (three independent experiments). * *p* < 0.05 compared with the M0 group, as calculated using a one-way ANOVA with post-hoc Tukey test. (**C**) COX-1/SDH-A protein (top panel) and mtDNA/nDNA (bottom panel) ratios in THP-1-derived polarized macrophages cultured for an additional four days in the presence or absence of 100 nM semaglutide and 200 μM SQ22536, with daily replacement of the corresponding culture medium. The COX-1/SDH-A ratio was determined using an in-cell ELISA assay. The mtDNA/nDNA ratio was determined by quantitative real-time PCR by comparing the level of the mtDNA-encoded MT-ND1 locus with that of the nuclear DNA-encoded reference gene B2M. For each sample, ΔCt was calculated as Ct (MT-ND1) − Ct (B2M). mtDNA/nDNA ratio is represented as the mean of 2^(−ΔCt)^. Data are presented as the mean ± SEM (five independent experiments). # *p* < 0.05 compared with the M1 group treated with semaglutide, as calculated using a one-way ANOVA with post-hoc Tukey test.

Polarization of both U-937- and THP-1-derived macrophages toward the M1 phenotype results in a significant reduction in expression levels of SIRT1 and PGC-1α, genes of the key regulators of mitochondrial biogenesis (Figure 3A). Semaglutide attenuates the M1-associated reduction in SIRT1 and PGC-1α expression, while inhibition of adenylyl cyclase by SQ22536 abrogates its effect, similarly to its effects on M1-/M2-associated markers and pro-/anti-inflammatory cytokines. Treatment with SQ22536 alone does not significantly affect PGC-1α expression compared with the untreated M1 group (Supplementary Figure S2C). Together, these findings suggest that semaglutide regulates the SIRT1/PGC-1α axis in a cAMP-dependent manner, raising the possibility that these transcriptional changes may result in modulation of the processes associated with mitochondrial biogenesis.

### 3.4. Semaglutide Induces cAMP-Dependent Changes in Mitochondrial Biogenesis-Associated Markers in THP-1-Derived Macrophages

To determine whether semaglutide-induced upregulation of SIRT1 and PGC-1α is accompanied by changes associated with mitochondrial biogenesis, THP-1-derived M1-like macrophages were cultured for an additional 4 days in the presence or absence of 100 nM semaglutide and 200 μM SQ22536. Mitochondrial changes were evaluated using two complementary approaches: the COX-1/SDH-A protein ratio and relative mitochondrial DNA content assessed as the ratio between the mtDNA-encoded MT-ND1 locus and the nuclear DNA-encoded B2M locus.

Because the prolonged experiment was performed after withdrawal of the initial LPS/IFN-γ stimulation, we tested whether an M1-associated transcriptional phenotype persisted during the extended cultivation period. Expression of the M1-associated markers CD80 and CD86 decreases compared with the initial 24-h polarization endpoint but remains elevated relative to M0-like macrophages (Figure 3B). Therefore, although the M1 inflammatory phenotype partially declines over time, THP-1-derived macrophages retain M1-associated transcriptional features throughout the experimental period.

As shown in Figure 3C, prolonged semaglutide treatment significantly increases the COX-1/SDH-A ratio compared to untreated M1-like macrophages. COX-1/SDH-A ratio reflects the relative abundance of the mitochondria-encoded respiratory complex IV subunit COX-1 and the nuclear DNA-encoded respiratory complex II subunit SDH-A and therefore represents an indirect marker associated with mitochondrial biogenesis [26]. Consistent with the COX-1/SDH-A results, semaglutide significantly increases the mtDNA/nDNA ratio compared with the untreated M1 group (Figure 3C). The addition of SQ22536 attenuates both ratios. SQ22536 alone has no significant effect compared with untreated M1-like macrophages. These results provide complementary evidence for mitochondrial adaptations consistent with enhanced mitochondrial biogenesis and support their dependence on cAMP signaling.

Neither SQ22536 nor semaglutide, alone or in combination, decreases metabolic viability of THP-1-derived macrophages compared with untreated M1-like cells under the experimental conditions (Supplementary Figure S3), indicating that the presence of SQ22536 or semaglutide does not lead to substantial loss of metabolically active cells.

## 4. Discussion

Macrophages are key drivers of chronic low-grade inflammation in metabolic diseases such as obesity and type 2 diabetes, specifically through tissue infiltration, pro-inflammatory cytokine release, and promotion of insulin resistance [27]. Macrophage activation is associated with metabolic reprogramming. Anti-inflammatory macrophages primarily get energy from fatty acid oxidation coupled with mitochondrial oxidative phosphorylation, while pro-inflammatory M1 macrophages predominantly rely on glycolysis [2]. Consequently, therapeutic interventions that restore mitochondrial homeostasis may supposedly affect macrophage metabolism and inflammatory activity. Semaglutide, a GLP-1RA widely used as an anti-diabetic medication, induces mitochondrial function in certain tissues [13,14,15], and its systemic anti-inflammatory action has been reported in many instances [28]. However, the link between these features, specifically in the context of macrophage activation, is poorly understood. In the present study, we used macrophages derived from two human monocytic cell lines to evaluate the direct action of semaglutide on the inflammatory polarization process.

GLP-1R couples predominantly to the stimulatory Gαs protein [10]. Its activation promotes adenylyl cyclase-mediated production of intracellular cAMP as a key transducer of downstream signals [11]. Semaglutide induces concentration-dependent cAMP accumulation in the M0- and M1-like macrophages differentiated from THP-1 and U-937 monocytic cells (Figure 1A). The concentration of semaglutide (100 nM) selected for the downstream experiments should be considered as the lowest tested concentration producing a near-maximal cAMP response in the in vitro macrophage models used rather than aiming to reproduce the clinically relevant exposure. According to the current product information, the expected steady-state plasma concentrations of semaglutide after the once-weekly maintenance doses of 2.4 and 7.2 mg are approximately 75 nM and 236 nM, respectively [29]. However, semaglutide extensively binds to plasma albumin, and the circulating unbound fraction is therefore considerably lower [29]. At the same time, direct comparison between unbound plasma concentrations and nominal concentrations used in cell culture should be made cautiously, since semaglutide also interacts with serum proteins in culture medium. Therefore, our downstream experiments should be primarily interpreted as a mechanistic study conducted under near-maximal GLP-1R activation conditions. The question of macrophage responses to lower semaglutide concentrations, including those within the range of expected clinical free drug exposure, requires additional analysis.

Importantly, GLP1R expression increases following macrophage differentiation, with reproducible detection of GLP1R mRNA in both M0- and M1-like macrophages (Figure 1B). Furthermore, pharmacological antagonism of GLP-1R with exendin(9–39) significantly attenuates semaglutide-induced cAMP accumulation (Figure 1C). Together, these results support the presence of functional GLP-1R signaling in the macrophage models used and indicate that GLP-1R contributes to the cAMP response elicited by semaglutide. Our findings should be considered in the context of apparently divergent observations regarding GLP-1R signaling in myeloid cells. Wong et al. showed that acute anti-inflammatory effects of GLP-1R agonism in mice were mediated mainly through neuronal rather than hematopoietic GLP-1Rs [8], whereas Wang et al. reported a direct effect of semaglutide on tumor-associated macrophages [9]. These differences may reflect variation in species, macrophage origin, and inflammatory context. Our results support functional GLP-1R signaling in THP-1- and U-937-derived macrophages, but should not be generalized to all macrophage populations.

Treatment with SQ22536, a potent adenylyl cyclase inhibitor [22], reduces semaglutide-induced cAMP accumulation, indicating attenuation of downstream GLP-1R signaling.

We demonstrate that semaglutide inhibits the pro-inflammatory response triggered by LPS/IFN-γ in M0-like macrophages. It attenuates the upregulation of the M1-like marker genes CD80 and CD86 [30] and reduces the secretion of TNF-α and IL-6 (Figure 2). Simultaneously, the treatment promotes expression of the M2-like marker genes CD206 and CD163 [30] and increases secretion of the anti-inflammatory cytokine IL-10. Together, these findings indicate attenuation of the inflammatory M1-like program accompanied by induction of transcriptional and functional features associated with a less inflammatory macrophage state. The ability of SQ22536 to attenuate the effects of semaglutide on the tested pro- and anti-inflammatory markers, together with the absence of significant effects of SQ22536 alone on representative endpoints, supports the involvement of cAMP signaling in macrophage responses to semaglutide.

A noteworthy observation of the current study is the ability of semaglutide to increase the expression levels of SIRT1 and PGC-1α genes disturbed as a result of pro-inflammatory polarization (Figure 3). Both SIRT1 and PGC-1α form a central regulatory axis linking cellular energy status, mitochondrial function, and inflammation [31]. PGC-1α is a transcriptional co-activator serving as the key master regulator of mitochondrial biogenesis and coordinating the expression of genes involved in oxidative phosphorylation, fatty acid β-oxidation, and mitochondrial DNA replication [32]. In turn, SIRT1 directly activates PGC-1α via deacetylation, thereby enhancing its transcriptional activity and establishing a positive regulatory loop that links the energy status of the cell to mitochondrial response [33]. Thus, the restoration of SIRT1 and PGC-1α levels potentially may reflect the reactivation of a suppressed mitochondrial biogenesis program and a shift toward a more oxidative metabolic profile, characteristic of a less inflammatory macrophage state. This is consistent with the known ability of GLP-1RAs to improve mitochondrial function, reduce oxidative stress, and enhance cellular resilience against damage [34]. Additionally, we established that long-term treatment of M1-like macrophages with semaglutide increases the COX-1/SDH-A ratio and relative mtDNA content (Figure 3C). Both COX-1/SDH-A [26] and mtDNA/nDNA [20] ratios are considered indirect indicators of mitochondrial changes associated with biogenesis. Therefore, our findings suggest that semaglutide promotes processes associated with mitochondrial biogenesis during macrophage M1-like polarization, potentially through upregulation of the SIRT1/PGC-1α axis.

Importantly, inhibition of adenylyl cyclase with SQ22536 attenuates mitochondria-associated effects produced by semaglutide (Figure 3), which correlates with data on M1- and M2-associated markers (Figure 2) and indicates the dependence on cAMP signaling. These findings suggest a link between semaglutide-induced cAMP accumulation, regulation of the SIRT1/PGC-1α axis, and macrophage polarization. AMPK represents a plausible intermediate mechanism connecting these processes. It is known that AMPK is a participant in the cAMP-initiated pathway [35], and in the case of GLP-1R signaling it is reported to be the direct effector of cAMP/PKA- and cAMP/EPAC-mediated cascades [36]. In the context of mitochondrial biogenesis, AMPK is shown to activate SIRT1 and PGC-1α [37] forming a major cellular network that senses energy levels, manages metabolism, and controls mitochondrial health [38]. Although AMPK activity was not directly assessed in the present study, we hypothesize that the cAMP–AMPK–SIRT1/PGC-1α signaling axis may represent a putative mechanism underlying the observed effects of semaglutide. However, the involvement of this pathway requires direct experimental validation in future studies.

The limitations of the present study should be acknowledged. In our experiments, we used macrophages differentiated from human monocytic THP-1 and U-937 cell lines. While these cell models allow for obtaining consistent and reliable results, they cannot fully recapitulate the phenotypic, metabolic, and functional heterogeneity of primary human macrophages. Moreover, macrophages may respond differently to semaglutide depending on their tissue of origin and the state of activation. Therefore, future studies aimed at confirming the physiological relevance of our findings should include primary human monocyte-derived macrophages, particularly those obtained from individuals with obesity, metabolic syndrome, or type 2 diabetes. Validation in appropriate in vivo models will also be necessary to determine whether the observed cAMP-dependent anti-inflammatory and mitochondrial responses contribute to the systemic effects of semaglutide in the context of chronic metabolic inflammation.

## 5. Conclusions

The present study demonstrates that semaglutide attenuates LPS/IFN-γ-induced inflammatory polarization in macrophages derived from human monocytic cell lines and promotes a shift toward a less inflammatory phenotype. These effects are associated with increased expression of SIRT1 and PGC-1α and are attenuated by inhibition of adenylyl cyclase with SQ22536, supporting an important contribution of cAMP signaling to the observed responses. In THP-1-derived macrophages, semaglutide also increases the COX-1/SDH-A ratio and relative mtDNA content, providing complementary evidence of mitochondrial adaptations consistent with enhanced mitochondrial biogenesis. Given the key role of mitochondrial dysfunction in metabolic inflammation, our findings suggest that the anti-inflammatory effects of semaglutide may involve the restoration of mitochondrial homeostasis and thereby may help explain the beneficial effects of GLP-1RAs in the context of chronic metabolic inflammation.

## Data Availability

The data presented in this study are available within the article and its Supplementary Materials. Raw data supporting the findings of this study are available from the corresponding author upon request.

## Supplementary Materials

The following supporting information can be downloaded at: https://www.mdpi.com/article/10.3390/biomedicines14091987/s1, Table S1: Primer sequences used for quantitative real-time PCR; Figure S1: LPS/IFN-γ-induced polarization does not cause statistically significant changes in IL-1β secretion in either THP-1-or U-937-derived macrophages. THP-1 and U-937 cells were differentiated into M0-like macrophages by PMA treatment and then polarized into the M1-like phenotype by incubation with 10 ng/mL LPS and 20 ng/mL IFN-γ for 24 h in the presence or absence of 100 nM semaglutide and 200 μM SQ22536. IL-1β concentrations in conditioned medium were measured by ELISA. Data are presented as the mean ± SEM (five independent experiments). Statistical significance was calculated using a one-way ANOVA with post-hoc Tukey test. Figure S2: SQ22536 treatment alone does not significantly alter the evaluated parameters compared with untreated M1-like macrophages derived from THP-1 and U-937. THP-1 and U-937 cells were differentiated into M0-like macrophages by PMA treatment and then polarized into the M1-like phenotype by incubation with 10 ng/mL LPS and 20 ng/mL IFN-γ for 24 h in the presence or absence of 100 nM semaglutide, 200 μM SQ22536, or their combination. Untreated M1-like macrophages were used as the control. (A) Relative mRNA expression of the M1-associated gene CD80 and M2-associated gene CD206. (B) TNF-α and IL-10 concentrations in conditioned medium measured by ELISA. (C) Relative mRNA expression of PGC-1α. Relative mRNA expression was determined using the comparative Ct method and normalized to ACTB. The mean ΔCt of the corresponding untreated M1 control group. Relative expression is expressed as 2^(−ΔΔCt)^, with the mean value of the untreated M1 group set to 1. Data are presented as the mean ± SEM (three independent experiments). * *p* < 0.05 comparedwith the untreated M1 group, as calculated using a one-way ANOVA with post-hoc Tukey test. Figure S3: MTT assay in THP-1-derived macrophages after polarization into the M1-like phenotype by incubation with LPS/IFN-γ for 24 h (M1 24 h) in the presence or absence of 100 nM semaglutide, 200 μM SQ22536, or their combination, and after culturing the cells for additional 4 days after polarization in the presence of the respective compounds and in the absence of LPS/IFN-γ (M1 4 days post-pol). The absorbance of the MTT formazan was determined at 570 nm using ELISA reader. Cell viability was defined as the ratio (expressed as a percentage) of absorbance of M1-like cells to M0-like cells. Viability of M0-like macrophages is expressed as 100%.

## Abbreviations

The following abbreviations are used in this manuscript:

ACSL1	Acyl-CoA Synthetase Long-Chain Family Member 1
ATP	Adenosine triphosphate
cAMP	Cyclic AMP
cDNA	Complementary DNA
COX-1	Cytochrome C oxidase subunit 1
ELISA	Enzyme-linked immunosorbent assay
Gαs	Stimulatory G protein alpha subunit
GLP-1	Glucagon-like peptide-1
GLP-1R	Glucagon-like peptide-1 receptor
GLP-1RA	Glucagon-like peptide-1 receptor agonist
GPCR	G protein-coupled receptor
HTRF	Homogeneous time-resolved fluorescence
IFN-γ	Interferon gamma
IL-1β	Interleukin-1 beta
IL-6	Interleukin-6
LPS	Lipopolysaccharide
mtDNA	Mitochondrial DNA
MTT	Thiazolyl blue tetrazolium bromide
nDNA	Nuclear DNA
PBS	Phosphate-buffered saline
PMA	Phorbol 12-myristate 13-acetate
PPARG	Peroxisome proliferator-activated receptor gamma
RT-PCR	Reverse transcription polymerase chain reaction
SDH-A	Succinate dehydrogenase subunit A
SEM	Standard error of the mean
STR	Short tandem repeat
TNF-α	Tumor necrosis factor
