# Supplementary material for: Semaglutide Attenuates Inflammatory Macrophage Polarization and Promotes Changes Associated with Mitochondrial Biogenesis Through cAMP-Dependent Signaling
Source: Biomedicines. 2026 Sep 3;14(9):1987. doi: 10.3390/biomedicines14091987 (PMC13604756; doi:10.3390/biomedicines14091987)
Supplement: Supplementary file 1 [file biomedicines-14-01987-s001.zip › biomedicines-4515822-supplementary.pdf]

Supplementary table S1

Primers for real-time RT-PCR	
β-actin fw	TGCGTGACATTAAGGAGAAG
β-actin rv	GTCAGGCAGCTCGTAGCTCT
CD80 fw	GAAATGTCGCCTCTCTGAAGA
CD80 rv	TGGATTTAGTTTCACAGCTTGC
CD86 fw	CTGCTGCTGTAACAGGGACT
CD86 rv	TCAGAGGAGCAGCACCAGA
CD206 fw	TTCCTTTGGACGGATGGACG
CD206 rv	GTCAAGGAAGGGTCGGATCG
CD163 fw	ACAGCGGCTTGCAGTTTCCT
CD163 rv	TGGCCTCCTTTTCCATTCCAGA
SIRT1 fw	TGGCACAGATCCTCGAACAA
SIRT1 rv	TGAAACAGACACCCCAGCTC
PGC-1α fw	GGAGTGACATCGAGTGTGCTG
PGC-1α rv	ACCACTTGAGTCCACCCAGA
GLP1R fw	CTACGCACTCTCCTTCTCTGCT
GLP1R rv	CGGACAATGCTCGCAGGATGAA
Primers for real-time PCR	
MT-ND1 fw	ACGCCATAAAACTCTTCACCAAAG
MT-ND1 rv	GGGTTCATAGTAGAAGAGCGATGG
B2M fw	CACTGAAAAAGATGAGTATGCC
B2M rv	AACATTCCCTGACAATCCC

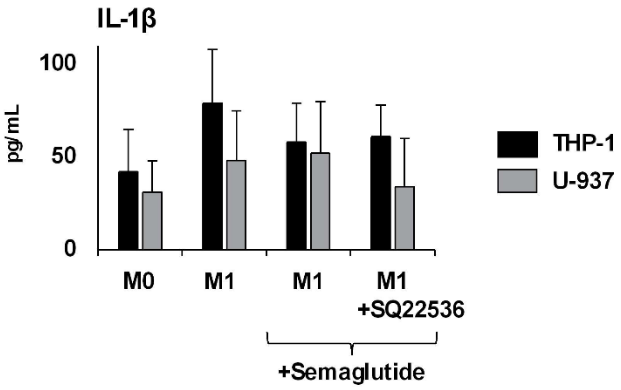

Figure S1. LPS/IFN-γ-induced polarization does not cause statistically significant changes in IL-1β secretion in either THP-1- or U-937-derived macrophages. THP-1 and U-937 cells were differentiated into M0-like macrophages by PMA treatment and then polarized into the M1-like phenotype by incubation with 10 ng/mL LPS and 20 ng/mL IFN-γ for 24 h in the presence or absence of 100 nM semaglutide and 200 μM SQ22536. IL-1β concentrations in conditioned medium were measured by ELISA. Data are presented as the mean ± SEM (five independent experiments). Statistical significance was calculated using a one-way ANOVA with post-hoc Tukey test.

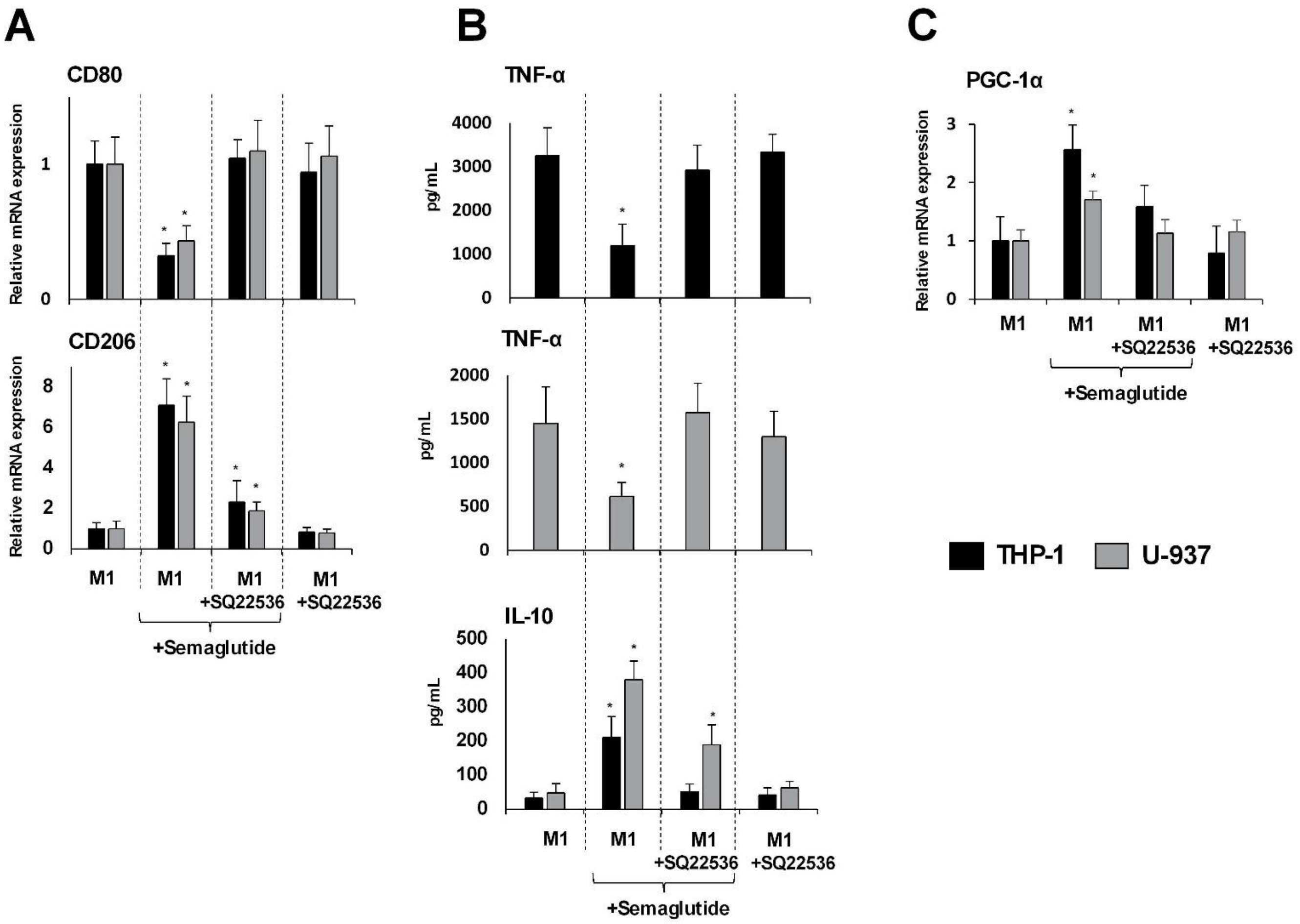

Figure S2. SQ22536 treatment alone does not significantly alter the evaluated parameters compared with untreated M1-like macrophages derived from THP-1 and U-937. THP-1 and U-937 cells were differentiated into M0-like macrophages by PMA treatment and then polarized into the M1-like phenotype by incubation with 10 ng/mL LPS and 20 ng/mL IFN-γ for 24 h in the presence or absence of 100 nM semaglutide, 200 μM SQ22536, or their combination. Untreated M1-like macrophages were used as the control. (A) Relative mRNA expression of the M1-associated gene CD80 and M2-associated gene CD206. (B) TNF-α and IL-10 concentrations in conditioned medium measured by ELISA. (C) Relative mRNA expression of PGC-1α. Relative mRNA expression was determined using the comparative Ct method and normalized to ACTB. The mean ΔCt of the corresponding untreated M1 control group. Relative expression is expressed as $2^{(-\Delta\Delta Ct)}$, with the mean value of the untreated M1 group set to 1. Data are presented as the mean ± SEM (three independent experiments). * $p < 0.05$ compared with the untreated M1 group, as calculated using a one-way ANOVA with post-hoc Tukey test.

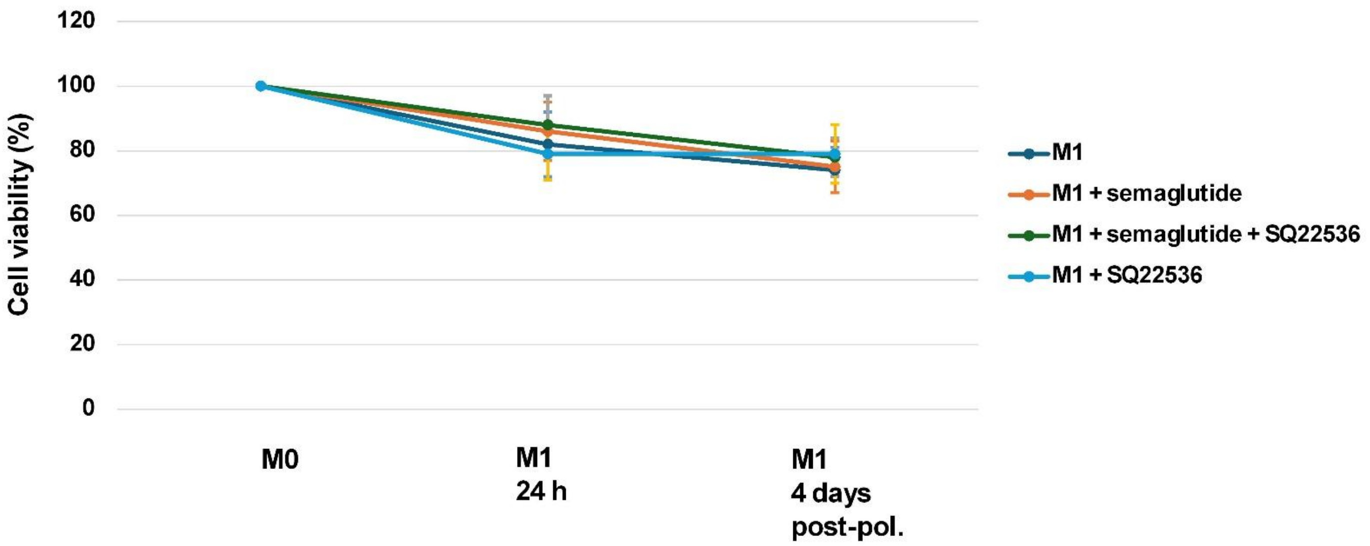

Figure S3. MTT assay in THP-1-derived macrophages after polarization into the M1-like phenotype by incubation with LPS/IFN-γ for 24 h (M1 24 h) in the presence or absence of 100 nM semaglutide, 200 μM SQ22536, or their combination, and after culturing the cells for additional 4 days after polarization in the presence of the respective compounds and in the absence of LPS/IFN-γ (M1 4 days post-pol). The absorbance of the MTT formazan was determined at 570 nm using ELISA reader. Cell viability was defined as the ratio (expressed as a percentage) of absorbance of M1-like cells to M0-like cells. Viability of M0-like macrophages is expressed as 100%.
